# Technical feasibility study for production of tailored multielectrode arrays and patterning of arranged neuronal networks

**DOI:** 10.1371/journal.pone.0192647

**Published:** 2018-02-23

**Authors:** Matthias Schürmann, Norman Shepheard, Natalie Frese, Kevin Geishendorf, Holger Sudhoff, Armin Gölzhäuser, Ulrich Rückert, Christian Kaltschmidt, Barbara Kaltschmidt, Andy Thomas

**Affiliations:** 1 Cell Biology, Bielefeld University, Bielefeld, Germany; 2 Department of Otolaryngology, Head and Neck Surgery, Klinikum Bielefeld, Bielefeld, Germany; 3 Center for Spinelectronic Materials and Devices, Physics Department, Bielefeld University, Bielefeld, Germany; 4 Cognitronics and Sensor Systems, Cognitive Interaction Technology Center of Excellence, Bielefeld University, Bielefeld, Germany; 5 Physics of Supramolecular Systems and Surfaces, Physics Department, Bielefeld University, Bielefeld, Germany; 6 Leibniz Institute for Solid State and Materials Research Dresden (IFW Dresden), Institute for Metallic Materials, Dresden, Germany; 7 Molecular Neurobiology, Bielefeld University, Bielefeld, Germany; Universita del Salento, ITALY

## Abstract

In this manuscript, we first reveal a simple ultra violet laser lithographic method to design and produce plain tailored multielectrode arrays. Secondly, we use the same lithographic setup for surface patterning to enable controlled attachment of primary neuronal cells and help neurite guidance. For multielectrode array production, we used flat borosilicate glass directly structured with the laser lithography system. The multi layered electrode system consists of a layer of titanium coated with a layer of di-titanium nitride. Finally, these electrodes are covered with silicon nitride for insulation. The quality of the custom made multielectrode arrays was investigated by light microscopy, electron microscopy and X-ray diffraction. The performance was verified by the detection of action potentials of primary neurons. The electrical noise of the custom-made MEA was equal to commercially available multielectrode arrays. Additionally, we demonstrated that structured coating with poly lysine, obtained with the aid of the same lithographic system, could be used to attach and guide neurons to designed structures. The process of neuron attachment and neurite guidance was investigated by light microscopy and charged particle microscopy.

Importantly, the utilization of the same lithographic system for MEA fabrication and poly lysine structuring will make it easy to align the architecture of the neuronal network to the arrangement of the MEA electrode.. In future studies, this will lead to multielectrode arrays, which are able to specifically attach neuronal cell bodies to their chemically defined electrodes and guide their neurites, gaining a controlled connectivity in the neuronal network. This type of multielectrode array would be able to precisely assign a signal to a certain neuron resulting in an efficient way for analyzing the maturation of the neuronal connectivity in small neuronal networks.

## Introduction

**Fig 1:**
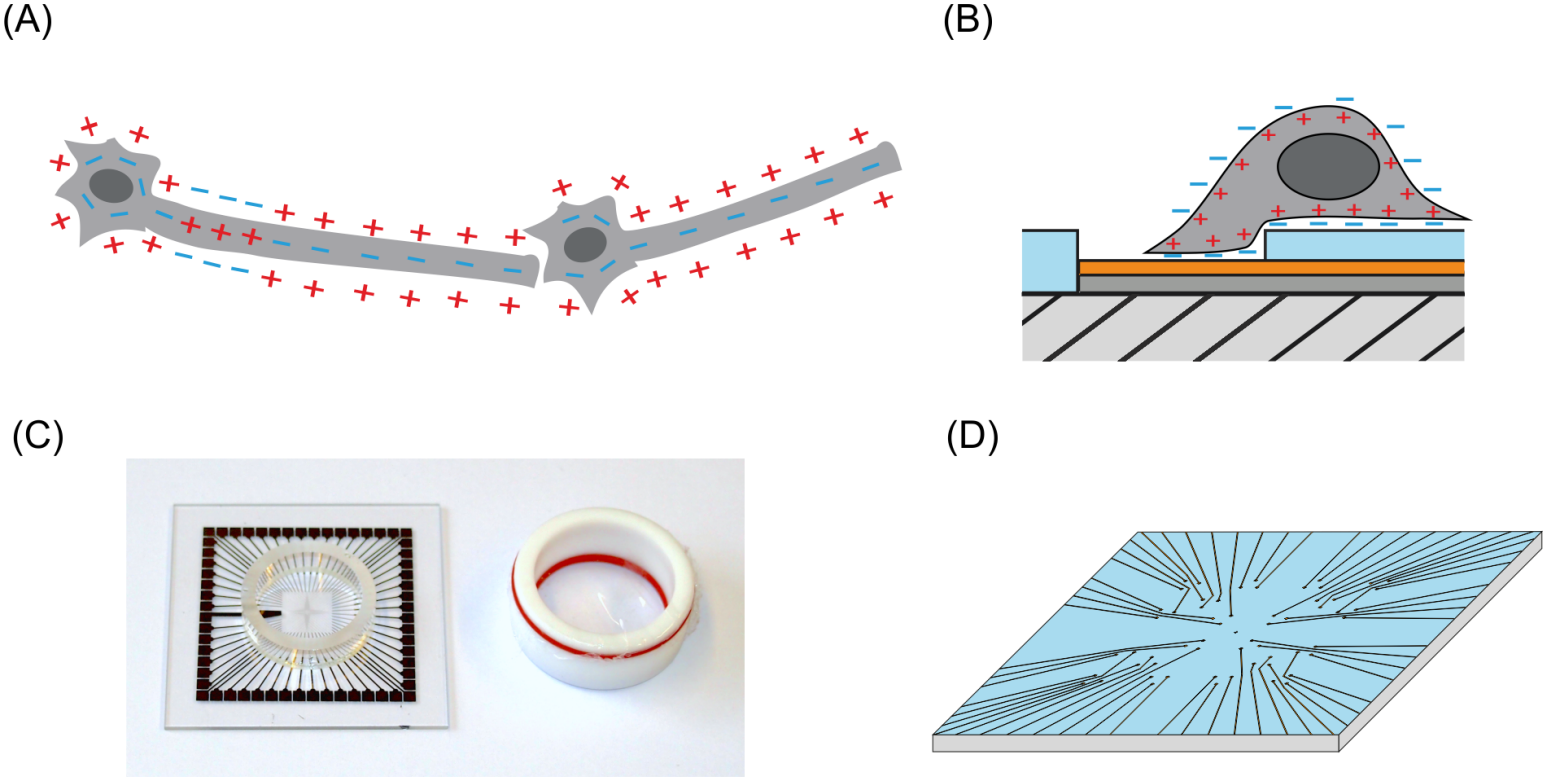
Schematic view on the multielectrode array technique. (A) Scheme of an action potential propagating along an axon of a neuron, which is connected to a neighboring neuron by a synapse. This composition represents the smallest unit of a neuronal ‘network’. (B) Detection of an action potential by a MEA electrode. The hatched area is the underlying glass substrate. The dark grey and orange layers show the conducting path, which consist of a titanium layer (dark grey) and a di-titanium nitride layer (orange). The top layer (blue) is made of silicon nitride serving as an insulator. A neuron, which partly covers the MEA electrode, is depolarized. Capacitive coupling to the electrode surface, which is tightly covered by the cell membrane, transmits the extracellular potential. (C) Image of the multielectrode array. The 59 contact pads lead to the measuring electrodes in the center and one reference electrode. For cell cultures, a glass ring is glued to the top. To prevent contamination, e.g. with bacteria or fungi, a lid is placed on the glass ring and sealed with an O-ring. (D) A scheme of the electrodes and the conductive pathway in the center of the MEA layout with a triangular geometry.

The characteristic feature of a neuron is its capability to generate and propagate action potentials. In this manner, these electrically excitable cells fulfill their main purpose by transmitting and processing information in the neuronal networks drawn through the organism (Fig 1A).

To study the mechanisms underlying the function of neurons, measurements of the action potentials from cells cultured in vitro can be carried out by various techniques, such as patch clamp or multielectrode arrays (MEAs). Patch clamp is a well-established technique, since Neher and Sakmann have introduced it in 1976 [1]. Even though there were many improvements introduced to this technique in the last decades, it still requires a lot of experience to be executed properly. Even though the patch clamp technique provides quantitative electrophysiological measurements of single cells or even ion channels, the measurements on many different neurons at exactly the same time is very difficult. In contrast, an analysis of several extracellular potentials from nerve cells in a network is possible with the MEA technique, which is easier to operate.

A bird´s eye view of a MEA is provided in Fig 1C, showing its general structure. Fig 1B schematically shows the function of a MEA with its core elements, the electrodes on top of the glass substrate and a neuron in close contact to the electrodes with a narrow gap between electrode and neuron. Titanium (Ti, dark grey) and di-titanium nitride (Ti_2_N, orange) are used here as the conductive materials for the electrodes. The blue layer is made of silicon nitride and insulates the conductive pathways from the majority of the cells. Even though the neuron is not in direct contact with the electrode, the extracellular potential in the gap between the electrode and the neuronal membrane can be probed by the electrode [2, 3]. In most cases, the neurons do not cover the whole electrode (Fig 1B). Nevertheless it is known, that the detectable signal is proportional to the ratio of its overlap with the electrode-area [2]. Since this ratio is not accessible in most cases, MEA measurements only give access to information regarding the location and frequency of action potentials in neuronal networks. To make the MEAs suitable for cell culture experiments, a glass ring is glued on to the top of the MEA (Fig 1C).

Although the MEA technique was first described by Thomas et al in 1972 [4], this technique was rarely utilized until the 1990s. This was mainly due to the lack of micro structuring facilities and computer power. Today, several advantages of the MEA technology come into effect.

A benefit is that MEAs receive the action potentials in a non-invasive way, which allows the experimenter to investigate the development of a neuronal network without disturbance. This option is used in pharmaceutical research, where the effect of substances on a neuronal network can be examined without affecting the cells in culture. Alongside to drug discovery, basic research on the growth and development of neuronal networks can be accomplished with the MEA technique as well, reviewed by Stett and coworkers [5]. Another remarkable advantage of MEAs is the simultaneous analysis of multiple electrodes or neurons, respectively. This is necessary to enable studies of the interaction within in neuronal networks. Hence, MEAs were applied to study the development of networks from neural progenitor cells [6], the properties of inhibitory or excitatory neurons within a network [7], the growth of functional connectivity [8], or even the controlled development of hippocampal networks on patterned substrates [9]. In addition to the investigation of neuronal networks developed *in vitro* MEAs are broadly used to study neuronal networks developed *in vivo* under *ex-vivo* conditions, like organotypic hippocampal slices [10, 11], or even intact tissue, e.g. retina as described in [12]. A further advantage of MEA technique is the possibility to utilize one arbitrary electrode for electrical stimulation of the neuronal network and simultaneously examine the reaction of the neuronal network upon stimulation. Based on the mentioned properties, the MEA is a very good technique to study the development of neuronal networks by electrical stimuli as demonstrated by Jewett *et al*. [13]. In summary, the MEA is a convenient tool to investigate the development of growing neuronal networks with the possibility to record and stimulate electric signals from cells.

One major disadvantage of a standard MEA is that the position of the electrodes is fixed and cannot be adapted to the architecture of a specific neuronal network. To overcome this problem, the electrode density can be adapted to increase spatial resolution. When pursuing this strategy, it should be taken into account, that the signal generated by the extracellular potential is captured from every cell in the range of the electrode diameter. Thus, the diameter of electrodes should be scaled down with an increase in electrode density. These high resolution MEAs are used to analyze retinal ganglion cells [14] or other dense populations. New complementary metal-oxide-semiconductor (CMOS) based MEAs improve this technology by overcoming the major problem for high density MEAs, which lies in the limited number of contacts to external devices [15, 16]. Today, multiplexing allows an electrode amount of 26400 electrodes [17]. The analysis of the large data from these CMOS MEAs is still a topic of research [18, 19].

Another more elegant approach to analyze neuronal networks might be to prompt the neuron soma to adhere directly to the electrodes. It is established, that an adhesion promoting surface coating, e.g., with poly lysine, is necessary to enable the attachment and survival of neurons in culture [20]. Hence, an adhesion promoting, dot-like structure on top of the MEA electrodes would be preferable.

Patterning of coated surfaces to control neuronal adhesion via lithographic technique was first realized by [21]. In addition to photolithography, different methods of surface patterning were developed. Corey and coworkers used laser ablation on poly lysine coated glass to locate neurons to a specific site [22]. Nowadays, the most common approach is the micro contact printing or soft lithography. During micro contact printing a polydimethylsiloxane stamp soaked with the cell adhesion molecule, is carefully pressed onto the sample surface [23]. More techniques are further described by Kane and colleagues [24]. Furthermore, structured poly lysine coating was already utilized to create artificial neuronal networks on MEAs [9]. This was accomplished with a rather coarse approach, not able to localize the cell soma to the electrodes.

In this study, we demonstrate a simple approach to produce tailored MEAs (cf. Fig 1D) with the aid of photolithographic devices and sputtering systems. Both devices are accessible as shared facilities of the science and engineering school in many universities and research institutions. Furthermore, we utilize the same lithographic device to create a structure of poly lysine, which is able to prompt the neurons somata to certain positions, and let their neurites extend in a systematic approach, which creates a defined network. The aim of this study is to determine the characteristics of a surface patterning regarding its geometry, which will support the localization of somata on the dots while preventing their localization on the connecting lines. Since we use the same laser lithographic device for structuring the MEA as well as the cell adhesive poly lysine pattern, an alignment of the electrode layout to the structure of the neuronal network can be easily accomplished. This kind of approach might answer one fundamental question in network simulations, e.g. the minimal required mechanism for one cell or synapse to work properly in neuronal networks.

## Materials and methods

### Multielectrode array fabrication

**Fig 2:**
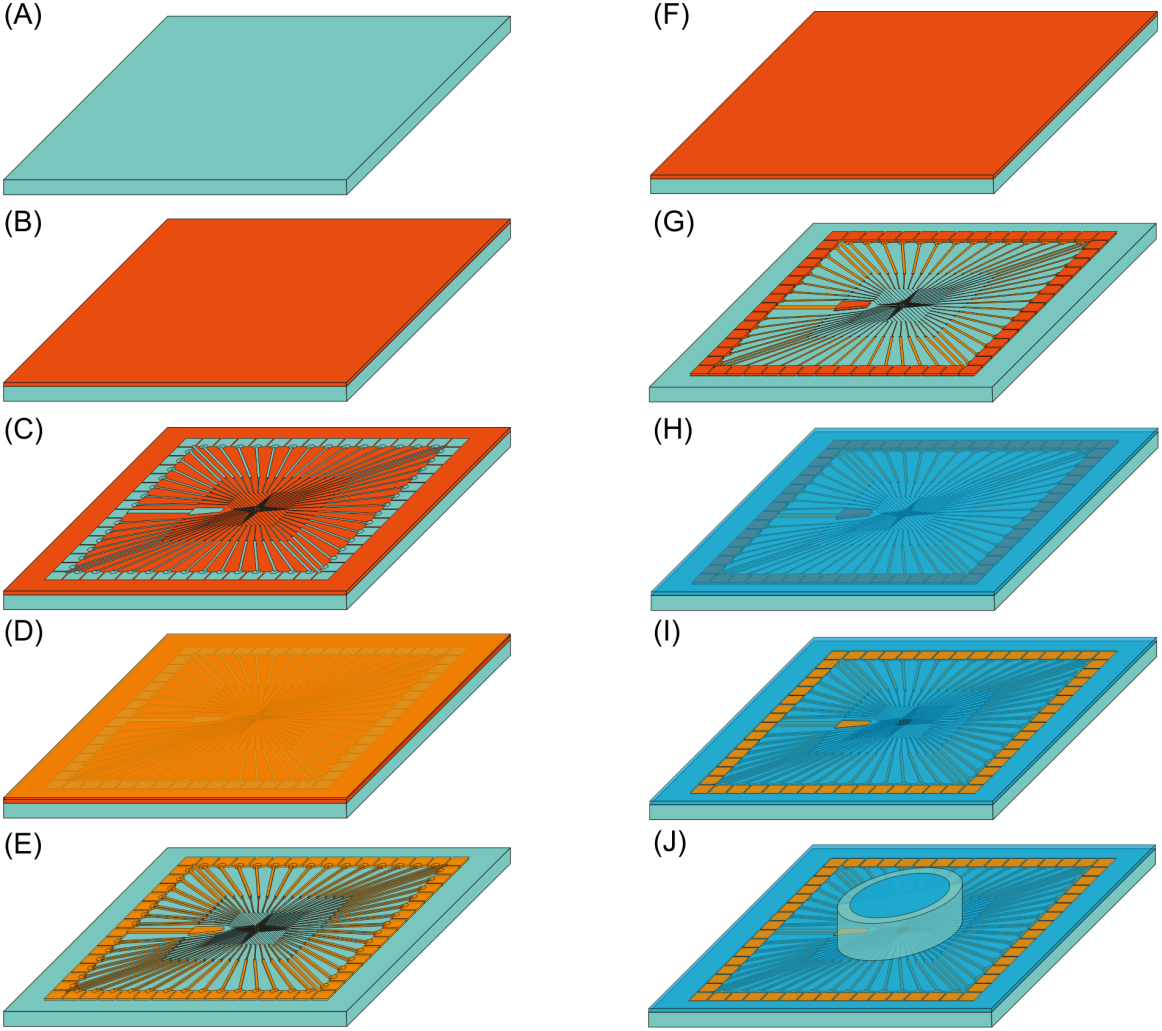
Fabrication strategy of our custom-made multielectrode array. (A) Cleaning of the glass substrate in acetone/ethanol in an ultrasonic bath. (B) Spin coating with positive photoresist, which is cured on a hot plate and then (C) exposed to a laser lithography system. Afterwards the exposed photoresist is removed with the dedicated chemical. (D) 100 nm thin layer of Ti is deposited via sputter deposition on the surface. Subsequently, a 25 nm layer of Ti_2_N is deposited on top. (E) The excessive photoresist is removed in an ultrasonic bath in acetone. (F) and (G) show a second lithography step, processed like the first one, except the photoresist remains solely on the contact pads on the rim and the electrodes in the center. (H) 200 nm of silicon nitride is deposited via sputtering on top. (I) The remaining photoresist is removed in an ultrasonic bath. (J) For cell culture experiment a glass ring is glued to the top. Thereafter, a second 25 nm thick layer of di-titanium nitride was reactively sputtered on top. The excess photoresist was lift off in an ultrasonic bath with acetone. The sample was cleaned with ethanol afterwards. The processed sample consists of the glass substrate with Ti/Ti_2_N electrodes (Fig 2E). To insulate the conducting paths, a second lithography step was necessary. The sample was resist coated as stated before (Fig 2F). This time, a pattern was obtained, which covered the electrode in the middle and the contact pads at the rim of the MEA (Fig 2G). The alignment of the second lithography step was done manually with a micro camera within the UV-lithography system, which allows an accurate alignment of the structures. After resist development, a 200 nm insulating layer of silicon nitride was deposited on top via RF sputtering (radio-frequency sputtering, Fig 2H) and the photoresist was lift off (Fig 2I). Finally, a glass ring was glued to the top with silicone aquarium sealant to make the MEA suitable for cell culture experiments (Fig 2J).

A square borosilicate glass (D263, Schott) with a side length of 49 mm and a thickness of 1.1 mm served as a substrate for the MEA. The glass was cleaned in an ultrasonic bath with acetone and subsequently with ethanol (Fig 2A). Afterwards, it was dried under a nitrogen stream. Positive Photoresist (AR-P 5350, AllResist) was spin-coated on the substrate for 60 seconds at 5000 rpm. In the following, the resist was cured on a hot plate for 4 minutes at 100°C (Fig 2B). The resist was exposed via a UV-laser lithographic system (DWL66, Heidelberg Instruments). The UV-lithography system was controlled via a computer aided design (CAD) file (.dxf file), which was drawn by a standard CAD program (AutoCAD). These exposed areas were dissolved with one part remover (AR 300–35, AllResist) diluted with two parts deionized H_2_O (Fig 2C). Afterwards the sample was rinsed with H_2_O for 30 seconds and dried with nitrogen. Subsequently, a 75 nm Ti layer was DC-sputtered on the surface via a self-build magnetron sputter deposition system (Fig 2D).

### Patterned poly lysine surface coating

A poly lysine pattern was applied on top of a glass surface. First, the surface was cleaned with acetone in an ultrasonic bath for 15 minutes. Secondly, the sample was immersed in ethanol for about 30 seconds. Afterwards, the cleaned surface was treated with oxygen plasma for 30 seconds (self-build plasma etcher). Subsequently, the whole surface was coated via gas phase coating with 3-aminopropyltriethoxysilane (APTES, Sigma-Aldrich). For this purpose, the sample and an upwardly open vessel with 200 μL APTES were placed in a desiccator for 1 h, which was evacuated below 10 mbar. For the correct assembly of the APTES layer, the sample was deposited for 24 h in a normal lab atmosphere at room temperature. Afterwards, the sample was prepared for lithography with photoresist (AR-P 5350, AllResist) on a spin coater (5000 rpm, 60 seconds) and structured by UV-laser-lithography. Following this, the resist was developed with one part AR 300–35 (AllResist) diluted with two parts deionized water. Finally, the sample was rinsed with deionized water and dried with nitrogen.

The sample surface, partly covered with the structured photoresist, was functionalized with a 6% glutaraldehyde (Sigma-Aldrich) solution in water. For this purpose, 300 μL of the solution was applied to the surface and the sample was stored at 4°C for 3 h. After washing with deionized water, the sample was coated with poly-d-lysine (PDL, MW = 1–5 kDa, Sigma-Aldrich) or fluorescein labeled poly-l-lysine (PLL-FITC, MW = 15–30 kDa, Sigma-Aldrich). For this purpose, 300 μL of a poly lysine solution (100 μg/ml in PBS, pH 8.0) was applied to the surface at 4°C for 16 h. Afterwards, the sample was washed several times with deionized water. Finally, the remaining photoresist was removed with acetone and ethanol in an ultrasonic bath. Because of this procedure, a checkered pattern with line widths of about 2.5 μm, 3 μm and 6 μm and dots of 30 μm diameters marking the nodes of the pattern were obtained (cf. S1 Fig).

### Cell culture

Mouse hippocampal neurons were isolated and cultivated as described in [25], with approval of the Landesamt für Natur, Umwelt und Verbraucherschutz NRW, Germany (LANUV NRW, No. 8.87–50.10.43.08.105). In particular, mouse hippocampal neurons were obtained by dissecting the hippocampi from the cortex of a mouse in the embryonic stage E17.5. The hippocampi were enzymatically digested for 30 min at 37°C in 3 ml of a solution containing 0.05% trypsin and 0.02% EDTA. Afterwards, the trypsin was quenched with 1 ml Dulbeccos modified eagle medium (DMEM) containing 10% fetal calf serum (FCS), and the tissue was broken down mechanically by gently pipetting through a Pasteur pipette. Subsequently, the cell number in the obtained suspension was determined. Thereafter, the cell suspension was further diluted in DMEM containing 10% FCS and the cells were plated on the samples with a density of 3*10^4^ cell/cm^2^. The medium was changed to neurobasal medium containing 1% B27 1 h, 24 h after seeding and subsequently every third day.

### Microscopy and sample preparation

Phase contrast images of living neuronal networks and structured collagen, and bright field images of MEA electrodes were obtained with a Zeiss Axiovert D1 microscope. The fluorescence imaging of the structured FITC-PLL coating was performed by confocal laser scanning microscopy (Zeiss LSM780). For the evaluation of the protein adsorption to the poly lysine pattern the substrates were incubated with a solution of DMEM containing 10% FCS and 1 μg/ml IgG Protein coupled with Alexa350 fluorophore at room temperature for 30 minutes. Afterwards, the samples were briefly rinsed with PBS, mounted with mowiol and examined using a confocal laser-scanning microscope (Zeiss LSM780). For immunocytochemistry, the cells were fixed with PBS containing 4% paraformaldehyde for 15 minutes. Subsequently, the neurons were permeabilized with 0.2% Triton X-100 in PBS for 10 minutes and incubated with a primary antibody against microtubule associated protein 2 (MAP2; rabbit, polyclonal, santa cruz, sc-20172) in phosphate buffered saline (PBS) at a concentration of 1 μg/ml at 4°C overnight. In the next step, the cells were incubated with PBS containing an appropriate secondary antibody coupled to Alexa647 at a concentration of 0.3 μg/ml at room temperature for 1 h. Finally, the slides were mounted with mowiol and examined using a confocal laser-scanning microscope (Zeiss LSM780). For helium ion microscopy (HIM) the samples were fixed in a solution of 4% glutaraldehyde and 4% paraformaldehyde in PBS at room temperature for 1 h. Subsequently, the samples were post-fixed with 1% osmiumtetroxide in PBS at room temperature for 30 minutes. After fixation, the samples were dehydrated in acetone and critical point dried via CO_2_. Imaging was performed with the helium ion microscopy ORION PLUS from Zeiss [26] with an acceleration of 39.9 kV and a beam current of 0.2 pA. An electron flood gun was used to compensate the charging of the uncoated samples.

### XRD and SEM

For an analysis of the sputtered electrode materials, x-ray diffraction (XRD, X’Pert Pro MPD, Philips) as well as scanning electron microscopy (SEM, Helios NanoLab DualBeam, FEI) were used. A layer stack of Ti and Ti_2_N was sputtered on silicon, likewise to the conductive material for the MEA. The XRD reflexes are compared to calculated data from the ICSD (Inorganic Crystal Structure Database, Collection Code: 033715). To investigate the structure of the created layer stack, a fragment of the sample was severed to obtain a cross section image of the layers by SEM.

### Electrophysiological measurement

The electrophysiological measurement was done with a MEA preamplifier (MEA1060 amplifier, MCS Reutlingen), as well as an external power supply (PS40W, MCS Reutlingen). We grab our signal over the 64-channel signal divider (SD64, MCS Reutlingen) and record one channel at a time via a multimeter with internal data acquisition (Keithley 2701/e, Keithley Instruments). The acquired data was read out with the aid of self-programmed data acquisition software. Finally, the signal was processed by a data analyzing software (OriginPro 2016) and fast-fourier filtered through a high pass with a cut off at 55 Hz. During noise measurements we compared the results between the self-built MEAs and commercial available MEAs (60MEA200/30iR-Ti, MCS Reutlingen) having the same electrode composition, metric and geometry.

## Results

### MEA fabrication and design

**Fig 3:**
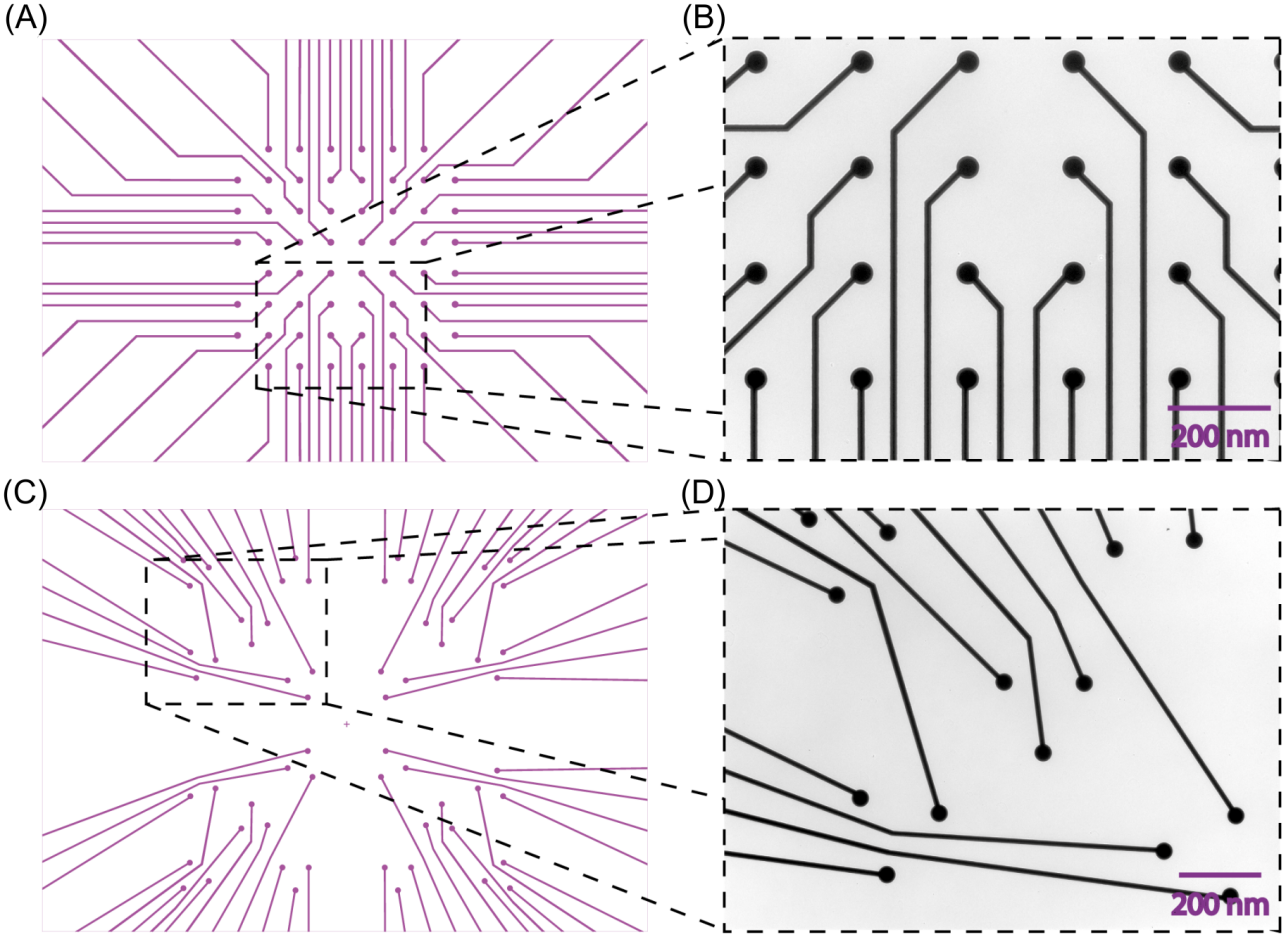
Design of multielectrode arrays. (A) and (C) show the central part of the CAD generated printout used to control the laser lithography system. The layout corresponds with the later conducting titanium and di-titanium nitride tracks with dot-like electrodes at the end. (A) Shows a standard rectangular layout with 200 μm distances between the electrodes. (B) The area in the black dashed box is shown as a bright field microscopic image of a MEA produced by this layout. Picture (C) is a custom-made design for a MEA. In this case, triangles with a base of 200 μm are used for the positioning of the electrodes. Image (D) shows the corresponding bright field microscopic image of the custom made MEA layout.

We fabricated MEAs in two different layouts, shown in Fig 3. We produced a standard layout often used in MEA set-ups with electrodes in a rectangular pattern. In Fig 3A, the CAD file for the first lithography step of a standard MEA layout with 59 electrodes is shown. The electrodes have 30 μm diameters and are spaced 200 μm from the neighboring electrodes. A conducting track links each electrode to one of 60 contact pads, which can be seen in Fig 1C. The one remaining contact pad is connected to the grounded reference electrode, needed to measure the potentials applied to the 59 measuring electrodes with respect to the ground. The conducting track is 10 μm wide and expands its width to 100 μm close to the contact pad. Fig 3B shows a microscopic image of the self-fabricated rectangular MEA. Fig 3C shows a MEA design based on a triangular layout. The baseline is 200 μm and the electrode diameter is 30 μm. Fig 3D shows a microscopic image of the produced triangular MEA. The flexibility to produce the proper MEA according to the experiment is a key feature of the CAD controlled laser lithographic system.

**Fig 4:**
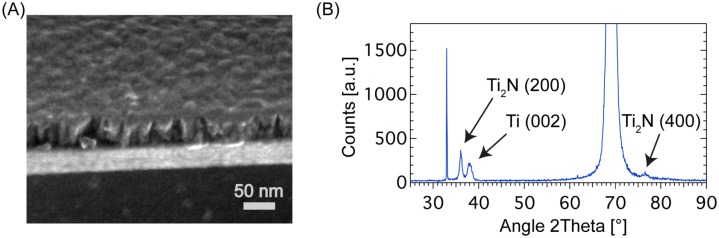
Electrode material characterization. (A) A SEM picture of a silicon substrate coated with layers of 40 nm titanium and 40 nm di-titanium nitride (Ti_2_N). The Ti_2_N crystals grow in characteristic columns. (B) X-ray diffraction (XRD) measurement from the same sample. Distinctive reflexes of Ti_2_N and the underlying Ti layer are detectable.

We used SEM for structure examination of the sputtered electrode layers, as well as XRD for analysis of the crystalline structure and the chemical composition of the two layers. The SEM image (Fig 4A) revealed a Ti_2_N layer, exhibiting its typical columnar morphology on top of the homogenous Ti layer. Further examination of the layer stack system by XRD (Fig 4B) revealed the first (200) reflex of Ti_2_N at 36.025° and the second (400) reflex at 76.425°. This is in accordance with the calculated reflectivity for tetragonal Ti_2_N (calculated from ICSD using POWD-12++, (1997)). The Ti layer showed reflexes at 38.175° representing the (002) orientation of Ti.

**Fig 5:**
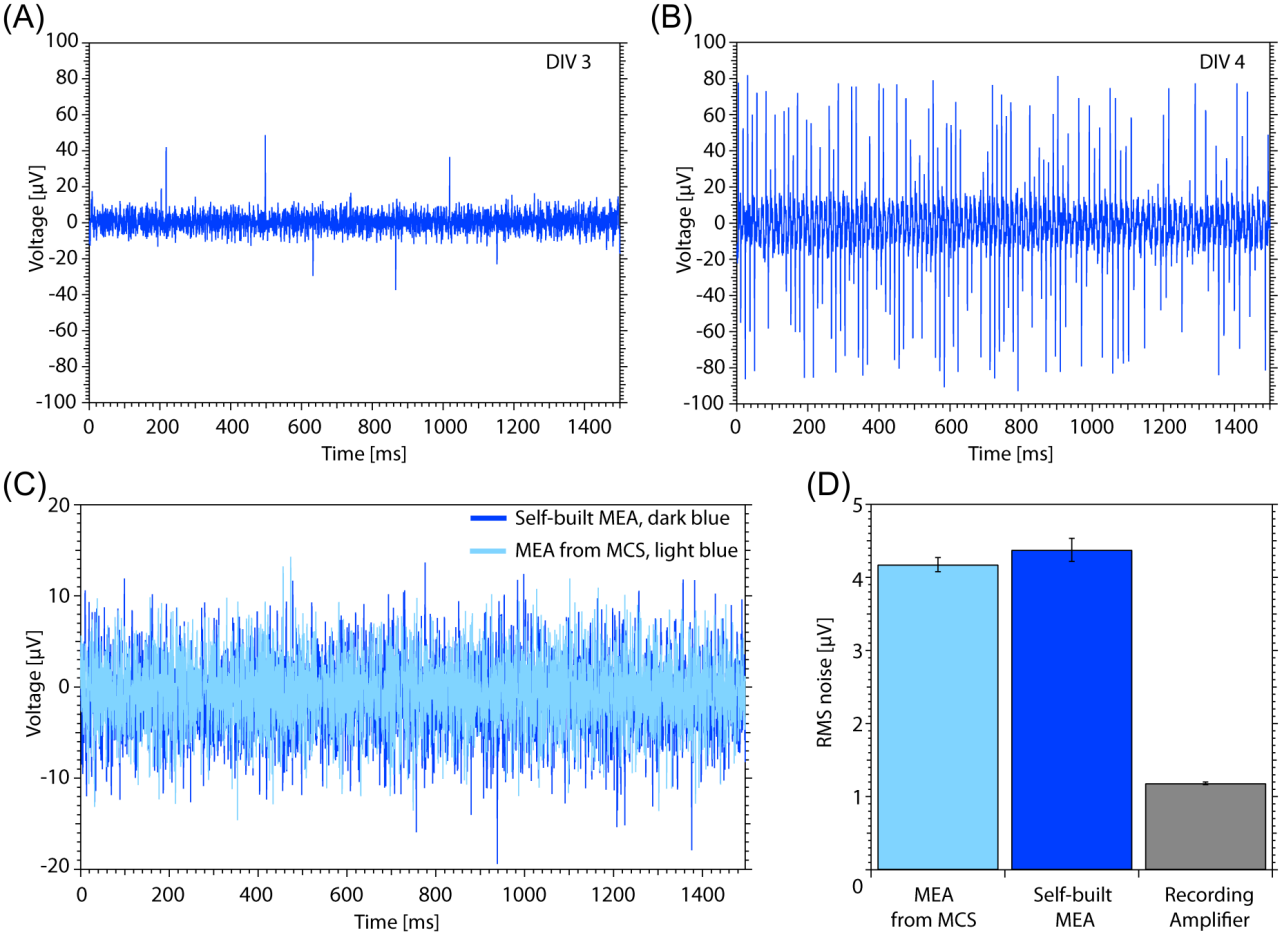
MEA measurements. (A) Measurement from a DIV3 neuron culture on the self-built MEA with rectangular layout show a few signals above the noise level. (B) A measurement on the same culture and electrode after DIV4. Accompanied with the progress in maturation, the spiking activity is visibly increasing. (C) Noise measurement of one electrode of the self-built MEA (dark blue) and a commercial available MEA from MCS, Reutlingen (light blue). (D) Comparison of the RMS noise values of the self-built MEA and the MEA from MCS (mean for 6 electrodes with standard deviation). The RMS noise of the self-built MEA is not significantly higher compared to the commercially available MEA from MCS. The noise measurement of the recording amplifier setup is below 1.2 μV and does not contribute significantly to the measured MEA noises.

To test the performance of our self-built MEAs, a neuronal culture from hippocampal mice neurons was cultured on the self-built rectangular MEA. The electrophysiological measurement in Fig 5A depicts a recording of the culture after 3 days *in vitro* (DIV3). Several spikes rise above the noise level with amplitudes of up to 40 μV. In Fig 5B, the same measurement performed after DIV4 is shown. The spiking activity is much higher than before. The noise measurement has been executed on plain MEAs without a neuronal culture, but with PBS in the culture chamber. In Fig 5C the noise of one electrode from our self-built MEA (dark blue) is compared to the commercially available one from the company MCS (light blue). We calculated the root mean square (RMS) noise for 6 arbitrary electrodes of each MEA and determined their mean (cf. Fig 5D). The commercially available MEA displays a RMS noise of 3.85 ± 0.09 μV, for our self-built MEA we calculated a RMS noise value of 4.04 ± 0.15 μV, demonstrating no significant difference to the commercially available MEA. The measured noise generated by the amplifying and recording setup does not contribute substantial to the overall noise.

### Structured poly lysine coating

**Fig 6:**
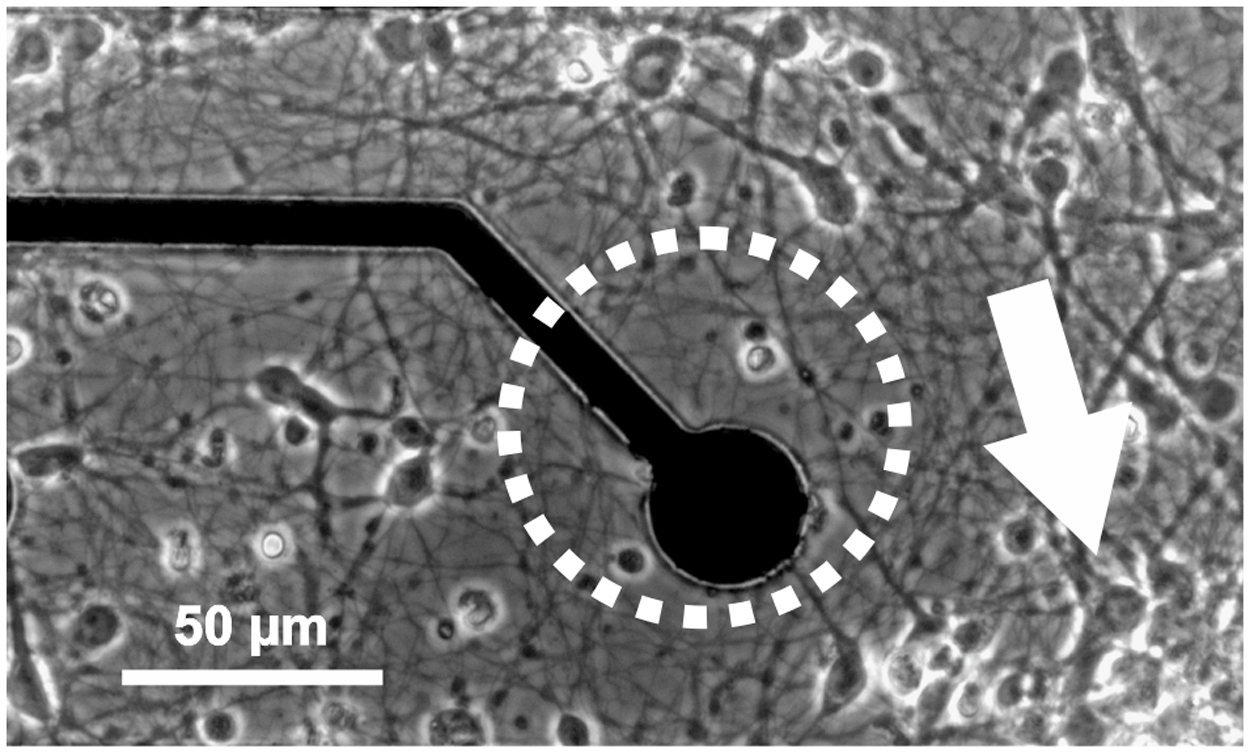
Hippocampal mouse neurons grown on custom made multielectrode array. Mouse neurons grown on a custom made MEA coated with PDL attach to the substrate in an anisotropic manner, with regions of higher (arrow) and lower (circle) cell density. Furthermore, the neurites exhibit no preferred direction of growth, resulting in an arbitrary neuronal connectivity.

Usually, the MEA is coated homogenously with molecules supporting the adhesion and maturation of neurons to the substrate, e.g. poly lysine or polyethylenimine (PEI). Fig 6 shows hippocampal mouse neurons grown on a custom made MEA coated with physically attached poly lysine. The cell density differs from region to region. In the dashed circle marks a low cell density area, at the arrow a cell cluster with high density can be observed.

**Fig 7:**
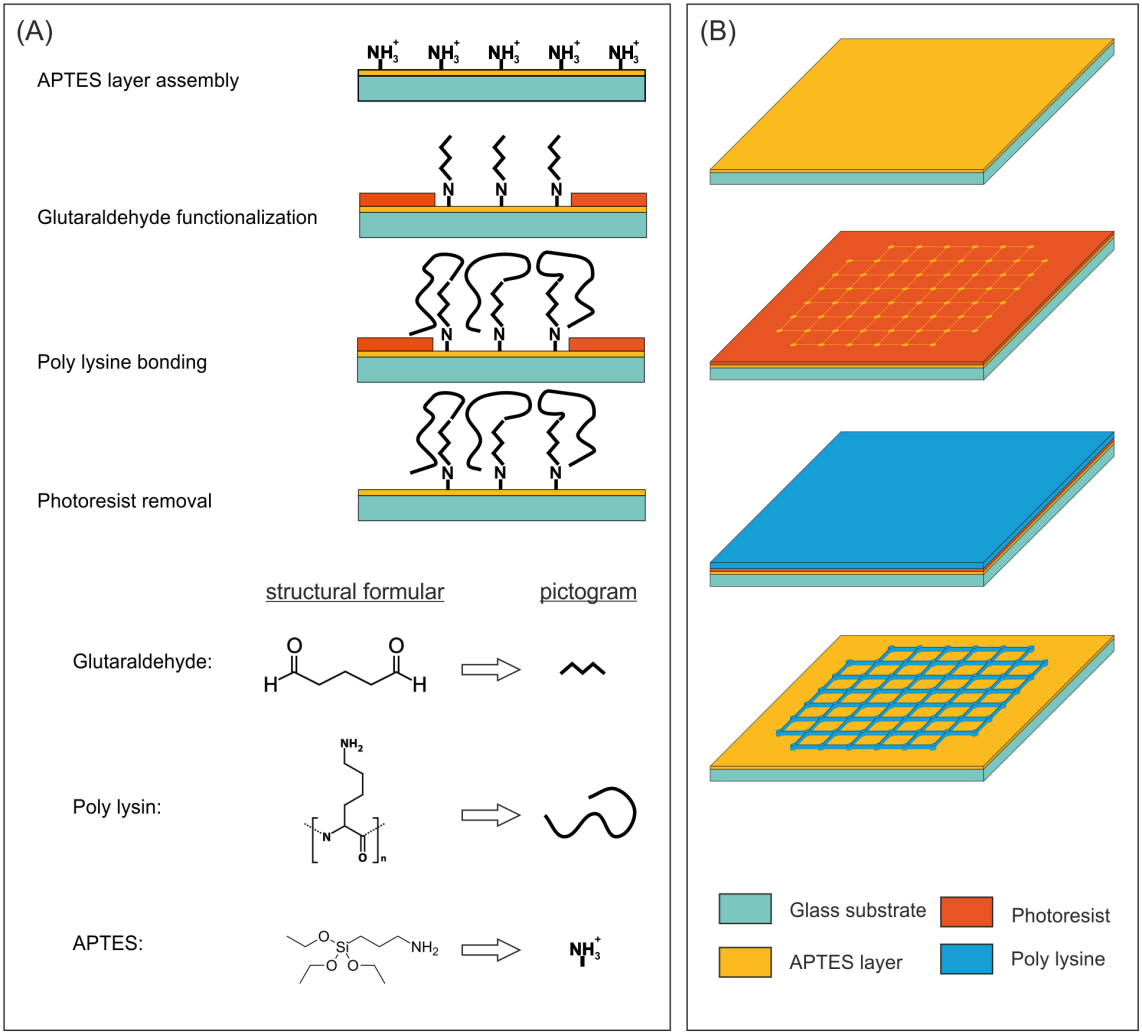
Poly lysine pattern fabrication for arranged neuron growth. (A) Provides the chemical process to gain a covalently bound poly lysine coating. The surface of the glass substrate is cleaned with acetone/ethanol in an ultrasonic bath. Afterwards the surface is etched with oxygen plasma to introduce hydroxyl groups to the surface. This surface is coated with a self-assembled layer of (3-Aminopropyl)triethoxysilane (APTES; yellow). After laser lithography, a layer of structured photoresist (orange) masks parts of the APTES layer. The next step is a treatment with glutaraldehyde to functionalize the surface. Following the washing of the sample with deionized water, the surface is treated with poly lysine and the sample is washed again. During this step, the poly lysine is covalently bound to the sample. Afterwards, the excess photoresist is removed with acetone in an ultrasonic bath. Finally, the substrate is decorated with a defined pattern made of poly lysine on an overall APTES grounding. (B) Schematically demonstrates the results of the chemical process for an applied pattern. After cleaning and plasma etching, the surface is coated with an APTES layer (yellow). Afterwards, the UV-laser lithography is performed; the orange layer is made of patterned photoresist. Glutaraldehyde and poly lysine are applied on top as described before. After washing the sample and removing the remaining photoresist with acetone in an ultrasonic bath, a pattern of covalently bound poly lysine remains on top of the sample.

The electrode seen in Fig 6 is not assigned to a single defined cell. A structured poly lysine coating as described in Fig 7 can be utilized to solve this problem. For this purpose, spots of lysine with a size allowing only one cell to adhere should be coated with poly lysine. In addition, small poly lysine lines are arranged between the spots, aiding the neurite guidance.

**Fig 8:**
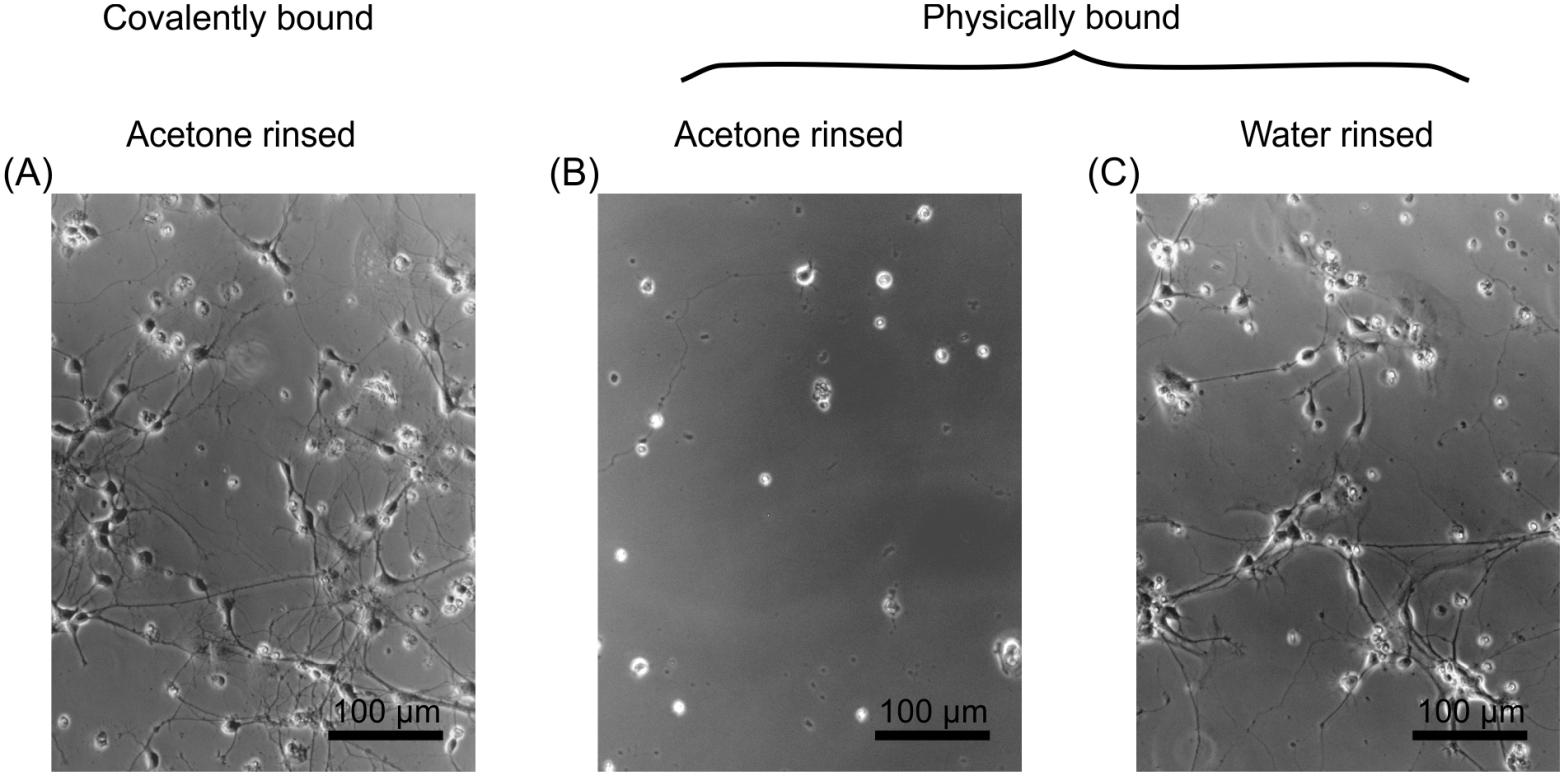
Hippocampal mouse neurons (DIV4) on differently coated substrates. (A) The substrate is furnished with covalently bound poly lysine. In (B) and (C) the poly lysine coating was solely physically adsorbed. The surfaces shown in (A) and (B) were cleaned in acetone in an ultrasonic bath while (C) was only rinsed with H_2_O. In contrast to (A) and (C), (B) shows no poly lysine attached to the substrate to promote the cell adhesion and neurite outgrowth. Therefore, a covalent attachment of poly lysine is necessary to obtain a structured poly lysine coating after acetone treatment during the lithographic process.

To understand the necessity of the covalent bonding of poly lysine, it must be taken into consideration that the coated sample has to be placed in acetone in an ultrasonic bath in order to remove the remaining photoresist (cf. Fig 7). Fig 8B shows a sample with poly lysine physically bound to the entire surface and cleaned in an ultrasonic bath in acetone. Compared to Fig 8C, where the same physically bound poly lysine surface is only rinsed with water, 8B shows nearly no adhered neurons on the sample, while the surface shown in 8C exhibits a well-developed neuronal network after DIV4. Hence, the physical bonding is not sufficient for an application in the utilized lithography process. To overcome this challenge, it is necessary to bind the poly lysine covalently to the sample surface (cf. Fig 8A).

To examine the quality of the surface coating, several imaging methods were utilized for verification. Initially, we obtained fluorescence images from the PLL-FITC coupled to the poly lysine (Fig 8A). The dot like structure was easily detectable and was measured to have a diameter of 30 μm, which is sufficient to promote the cell adhesion of a neuronal soma. Furthermore, thin lines of poly lysine connecting the single cell adhesion spots for neurite guidance have a width of approximately 2.5 μm.

**Fig 9:**
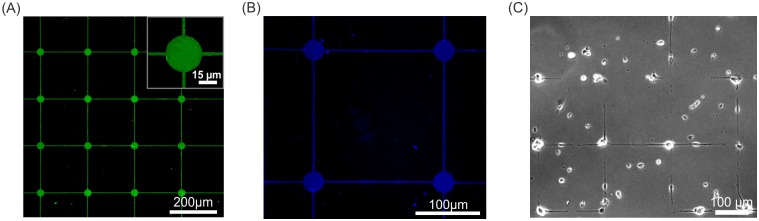
Covalent attachment of structured PDL is sufficient to induce an arranged attachment of neuronal cells and a directed outgrowth of their neurites. (A) Fluorescence microscopic image of structured poly lysine coupled to PLL-FITC. The homogenous distribution of the poly lysine is visualized as well as the 30 μm diameter of the dots and the 2.5 μm thickness of the lines connecting the dots (cf. inset). (B) Fluorescence microscopic image of the structured poly lysine coated surface with adsorbed fluorescently labeled proteins. The applied coating offers a much higher adhesiveness to proteins compared to the uncoated regions of the surface. (C) Phase contrast image of hippocampal mouse neurons grown for three days on the structured poly lysine coating. The neuronal somata tend to adhere to the circles marking the junctions of the checkered pattern, while the lines connecting these circles guide the neurites.

To verify the adhesion promoting characteristics of the substrates, we tested its protein adsorption properties. For this purpose, we incubated the patterned substrates with a solution containing fluorescently labeled proteins. The poly lysine pattern demonstrated a much higher protein adsorption capability compared to the overall APTES background (Fig 9B).

Finally, we tested the developed substrates in a cell culture experiment (Fig 9C). The neuronal culture showed a high degree of arrangement on the substrate. The majority of the cell somata were located on the junctions of the checkered pattern, where poly lysine spots of 30 μm diameter provided enough space to allow their cell soma to attach. On the other hand, the thin lines of poly lysine connecting the spots provided enough space for the neurites to develop their mature morphology.

**Fig 10:**
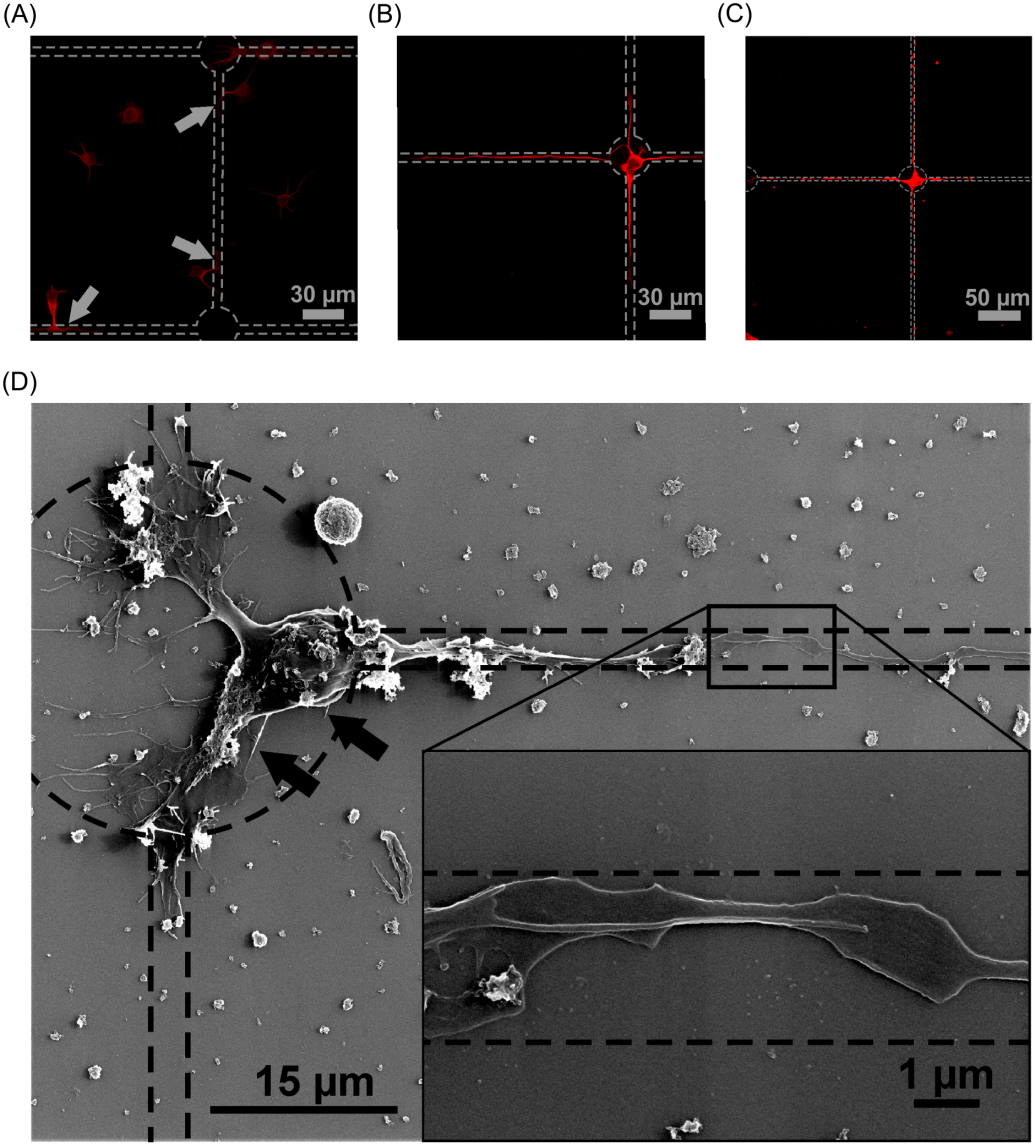
The processes involved in the controlled attachment and neurite outgrowth of the neurons on patterned substrate. Neurons are immuno-fluorescently labeled for the protein MAP2, a marker for neuronal maturation. The dashed lines indicate the poly lysine pattern. (A) 18 hours after cell-seeding the neurons have attached to the substrate in a rather arbitrary manner and are attracted by the poly lysine coated surface, notable by the protrusions attached to poly lysine (arrows). (B) After DIV3 the somata of the neurons are mainly located on the dots on the nodes of the checkered poly lysine pattern (cf. Fig 9). Furthermore, their neurites are well developed and the translation of MAP2 is upregulated. (C) Even after DIV8 the somata stayed attached to the nodes and the neurites were further elongated, reaching into the neighboring node (arrowhead). (D) HIM image of a neuron seeded on an equal poly lysine pattern. The tight adherence of the soma is visible by adhesion protrusions (arrows). Investigation of the guided axon (inset) elucidated that the axon is tightly bound to the substrate by well-developed lamellar protrusion (arrowheads).

We further investigated the development of the neuronal network on the patterned substrates. For this purpose, we fixed neurons 18 hours, 3 days and 8 days after cell-seeding and stained the cells for MAP2, a marker for neuronal maturation. It appeared that the neurons adhere to the substrate after initial attachment in a rather arbitrary manner (Fig 10A). However, they showed a high tendency to send out protrusion to the poly lysine pattern and allegedly started to migrate towards the coated surface. After three days in culture, most of the neurons had migrated towards the nodes at the junctions of the pattern (Fig 10B). The 30 μm dots seemed to be more attractive for the attachment of their soma compared to the thin lines with a width of about 3 μm. Presumably, the dots provided enough space to host the somata (which have a diameter of approximately 15 μm) without restricting its development in any direction (Fig 10D). Furthermore, the neurons showed an upregulation of MAP2 and a well-developed morphology of mature neurons with their neurites spanning over hundred micrometers. Even after DIV8 the soma of the neurons stayed attached firmly to the nodes, demonstrating the durability of the covalently bound coating. During the progress in neuronal maturation, the neurites further elongated to a length of up to 200 μm, reaching into the neighboring node.

**Fig 11:**
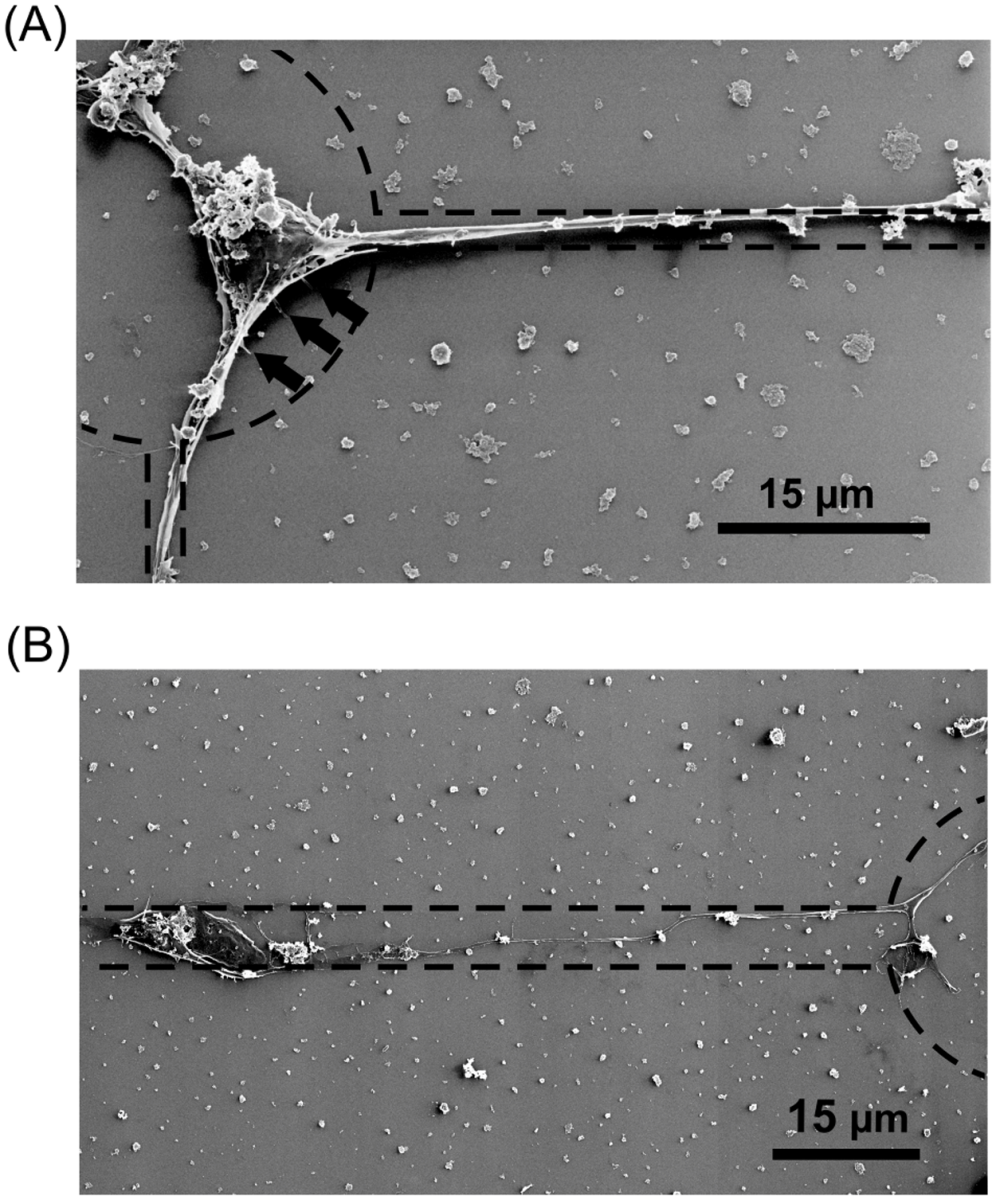
Line width dependence of neuronal soma adhesion to poly lysine lines. HIM image shows a neuron grown on lines with a width of about 2.5 μm (A) and 6 μm (B). The dashed lines indicate the poly lysine pattern. The neuron on the poly lysine pattern with thin line (A) is tightly adhered to the node of the checkered pattern as visualized by adherence promoting protrusion (arrows). In contrast to that the soma of a neuron adhered to a wider poly lysine line (B) stays fixed on the line and is not directed to migrate to the node of the checkered poly lysine pattern.

The localization of a soma on the node after DIV3 could also be demonstrated on similar pattern by HIM (Fig 10D). The soma showed a well-developed native morphology and adhered firmly to the poly lysine node as demonstrated by the filopodia-like protrusions. High resolution images of the axon grown along the poly lysine lines show a tight adherence of the neurite to the substrate. This is manifested by lamellar extension arising from the axon (cf. inset Fig 10D). Furthermore, HIM demonstrated that thick lines with a width of approximately 6 μm provide enough area for neurons to adhere (Fig 11B). The neuron can be localized either on the lysine node or the lysine line with an elongated soma, as shown in Fig 11B. This is in strong contrast to cells seeded on substrates with line widths of about 2.5 μm (Fig 11A), which prompted the soma to localize on the nodes, as already demonstrated.

## Discussion

We demonstrate an opportunity to equip MEAs with chemical coating to enable electrophysiological measurements on an arranged neuronal network. The presented method for customized production of MEAs using laser UV-lithography offers a row of advantages. Compared to standard contact lithography, no photomask is needed for the laser lithography. This is advantageous in terms of the processing rate of our MEA production process, since several lithographic applications are required and a manual alignment of different masks would otherwise be necessary during every step. Besides, laser lithography works on several length scales. The smallest printable feature is approximately 1 μm in size. The area printed during the first lithography step is about 39 mm by 39 mm. The major advantage is easy adaptability of the CAD controlled laser lithographic system used in this study. This enables an easy alteration and high flexibility of the MEAs layout. This is of outmost importance in basic research where experimental setup changes frequently. In this context, layouts with two different electrode arrangements and an electrode diameter of 30 μm with an electrode spacing of 200 μm were chosen. For example, further downscaling of the electrodes can easily be achieved with the described methods, but the electrode spacing should be altered simultaneously, with respect to the range of the received signals.

In our MEA design, we used a layered system of Ti and Ti_2_N as conductive materials. The biocompatible Ti_2_N is deposited on top of the Ti and serves as passivation. This is necessary, since titanium tends to oxygenate to amorphous titanium oxide with insulating characteristics to a depth of about 3nm [27]. This will result in non-uniform electrical properties of the surface, which will tend to change upon contact with cell culture media [28], making it difficult to compare signals from different electrodes. Besides its protective function, Ti_2_N is known to form a columnar microstructure, hence reducing the impedance of the electrodes. This will reduce their thermal noise in comparison to even Ti electrodes [29] and enhance charge transfer capability [30]. Silicon nitride is used as the insulating material on top of the MEA, its transparency making it suitable for microscopic investigation of the neurons cultured on the MEA. Furthermore, its hardness helps to protect the delicate conductive structure from scratches during cell culture processing. Electrophysiological measurements of the RMS noise from the self-built MEA and a commercial available MEA from Multichannelsystems shows no significant difference. Importantly, our electrophysiological measurements of cultivated neurons demonstrate the capability of the self-built MEA to distinguish between voltage peaks generated by action potentials and the overall noise. This is of particular interest, since the presented custom built MEAs cost around 10% of commercial available MEAs. Considering that many researchers nowadays have access to lithographic and sputtering devices, our approach offer a cost-effective alternative, which also can be tailored to the actual researcher needs.

The developed method for the patterning of adhesion molecules can be applied for direct attachment of neurons to the MEA electrodes. Buitenweg *et al*. demonstrated that the localization of neurons on top of the electrodes will lead to an increase in the signal to noise ratio [3]. More importantly, since only one neuron is in the receiving range of an electrode, an assignment of a signal to one specific neuron would be feasible. To achieve this key point, cage-like shapes on top of MEA electrodes were applied [31]. A far more convenient method is to apply a pattern made of molecules promoting the adhesion of cells on the electrodes. This method was realized by micro contact printing [9] or photolithography [32] with the aid of the well characterized adhesion molecule poly lysine. Our approach utilized UV-laser lithography in combination with the covalent binding of the poly lysine. The covalent binding will make the overall coating not only resistant to lithographic processing steps involving acetone (cf. Fig 8), but it will also allow the poly lysine structure to stay defined under cell culture conditions and more adhesive compared to physiosorbed poly lysine [32]. In our experiments, this could be demonstrated by long lasting confinement of the soma to the nodes (cf. Figs 10C and 11A). This covalent binding can also be achieved with different adhesion promoting molecules, as long as they contain amine groups, like PEI [33] or poly ornithine [34]. In addition, proteins that can withstand the denaturing conditions of the coating process, could be used as well, e.g. collagen (cf. S2 Fig), which had been long known for promoting neuronal adhesion [35]. With the localization of a single neuronal cell on top of an electrode, it should be possible to count the generated action potentials of this particular neuron. This is of great interest, when the metabolism of a single neuron needs to be studied, which can be measured by Matrix-assisted laser desorption/ionization time-of-flight (MALDI-TOF) mass spectroscopy on arrayed dead cells [36] or even on living cells *in vitro* by fluorescence microscopic means [37].

To study the development of a neuronal network, a second task has to be managed: the guidance of the neurites. The structured coating of covalently bound poly lysine was already utilized to create artificial neuronal networks [32]. In this study, the chosen line width was too big (20 μm) and hence no localization of the soma to the nod was observable. The structuring of proper poly lysine pattern with micro contact printing [38] has its difficulty, because it is complicated to produce lines thin enough to fulfill the mentioned needs. In the study of Boehler *et al*. from 2012, [9] the rather low resolution of the utilized micro contact printing, resulted in a line width of only 10 μm, which made it impossible to locate the neuronal soma on the nodes of the intersecting lines. We decided to use UV-laser lithography, because thin lines can be easily structured by this technique. Neurite guidance can be accomplished with 2.5 μm wide lines of adhesion molecules connecting the electrodes. The thickness of this structure must be balanced between large enough to ensure neurite growth and small enough to prohibit the adhesion of soma on the lines. We could observe that poly lysine lines with a thickness of 6 μm were not sufficient to force the soma to be located on the nodes. This finding is in accordance to previous studies [39] focusing on the same issue.

Furthermore, we established a technique to align the cultured neuron on a surface with the aid of structured cell adhesion molecules to make the MEA suitable to study the development of neuronal networks on a single cell level. In the study of Boehler *et al*. from 2012, micro contact printing was utilized to create artificial neuronal networks on MEAs [9], but the difficult positioning of the micro contact stamp prohibited an exact alignment of the poly lysine structure to the MEA [9, 40–47]. An advantage of the UV-laser lithographic structuring of the poly lysine pattern described in this paper is the feasible alignment of the adhesive pattern to an electrode array structured with the same lithographic device. Furthermore, the method presented in this study, the CAD file for the MEA can be reused offhand for structuring the poly lysine pattern with few modifications. Hence, the adhesion pattern can be designed to match each self-built MEA individually. This can be utilized in future studies to perform research on well-defined neuronal networks with directed neurite maturation. Further designs might match to the self-built triangular MEAs, so that defined neural logics of three neurons can be studied. To focus on the interplay in simple networks, understanding of the basic rules of large neuronal networks can be accomplished. In this manner, Pershin *et al*. [48] demonstrated the formation of associative memory with three electronic neurons. Similar experiments could be executed with living neurons *in vitro*.

Since the presented strategy of axon guidance is biocompatible and, due to the covalent bonding, long lasting, it could be applied to support directional growth of axons, which is crucial for peripheral nerve regeneration. Therefore, findings from this work could be extended to an *in vivo* situation to aid peripheral nerve regeneration after injury or to improve integration of electrodes from retinal implants or other neuroprosthetic devices.

## Supporting information

S1 Fig Structures with different line width. Confocal laser scanning microscopic image of PLL-FITC labeled poly lysine pattern. The three different line width of 6 μm (A), 3 μm (B) and 2.5 μm (C) are easily distinguishable.
(TIF)

S2 Fig Layer of covalently attached structured collagen is visualized by phase contrast microscopy. Phase contrast image of collagen covalently attached to the surface with the same pattern design used for the poly lysine coated samples. The procedure to fabricate this collagen sample follows the method for the patterned poly lysine coating except the use of collagen instead of poly lysine. Despite the collagen sample in the image was threatened with acetone in an ultrasonic bath during fabrication, the collagen pattern is still on top. The dots have 30 μm diameter and the connecting lines are 2.5 μm wide.
(TIF)
