# Supplementary figures and images for: Technical feasibility study for production of tailored multielectrode arrays and patterning of arranged neuronal networks
Source: PLoS One. 2018 Feb 23;13(2):e0192647. doi: 10.1371/journal.pone.0192647 (PMC5825013; doi:10.1371/journal.pone.0192647)

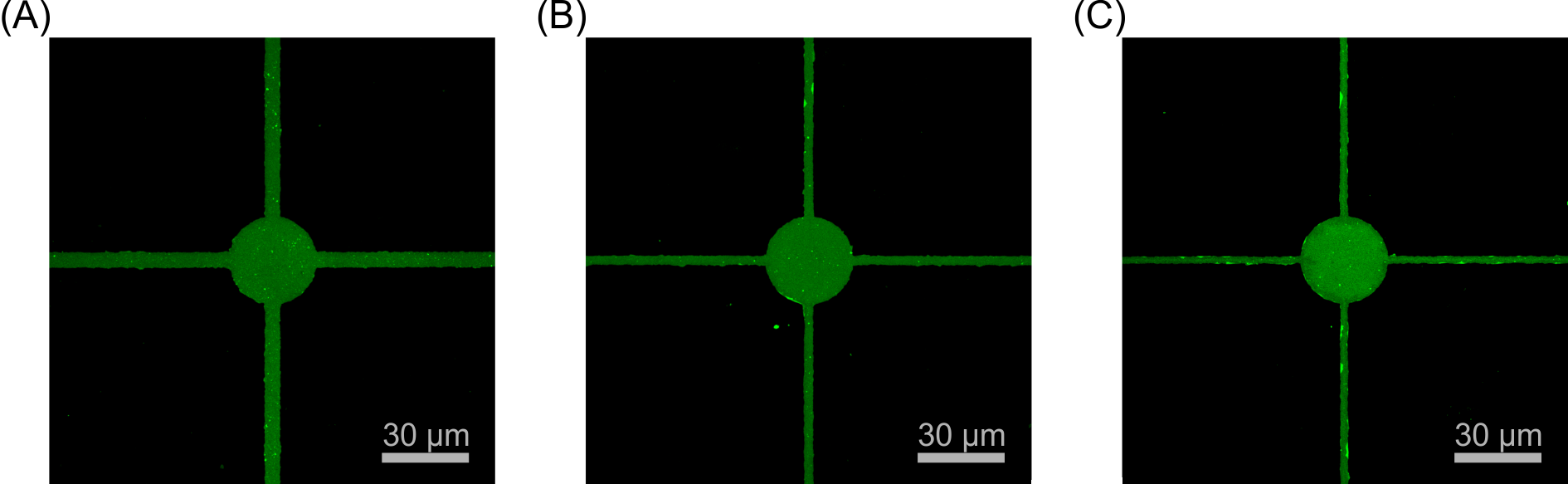

Supplement: S1 Fig — Confocal laser scanning microscopic image of PLL-FITC labeled poly lysine pattern. The three different line width of 6 μm (A), 3 μm (B) and 2.5 μm (C) are easily distinguishable. (TIF) [file pone.0192647.s001.tif]

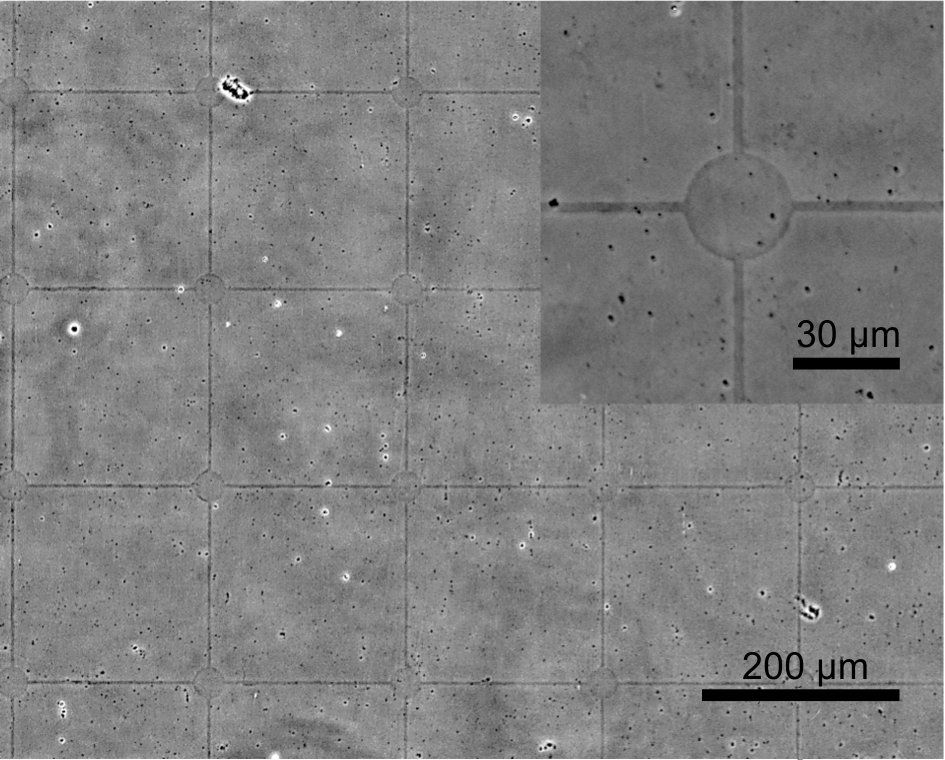

Supplement: S2 Fig — Phase contrast image of collagen covalently attached to the surface with the same pattern design used for the poly lysine coated samples. The procedure to fabricate this collagen sample follows the method for the patterned poly lysine coating except the use of collagen instead of poly lysine. Despite the collagen sample in the image was threatened with acetone in an ultrasonic bath during fabrication, the collagen pattern is still on top. The dots have 30 μm diameter and the connecting lines are 2.5 μm wide. (TIF) [file pone.0192647.s002.tif]
